# Who's in the Game? Cross‐Sectional Analysis of Sport Participation in Youth With and Without Disabilities

**DOI:** 10.1111/cch.70363

**Published:** 2026-10-15

**Authors:** Natalie H. Stagnone, Andrew Parks, Travis E. Dorsch, Jordan A. Blazo, Tom Farrey, Jon Solomon, Jennifer Brown Lerner

**Affiliations:** ^1^ Elson S Floyd College of Medicine Washington State University Spokane Washington USA; ^2^ Department of Kinesiology Louisiana Tech University; ^3^ Department of Human Development and Family Studies Utah State University; ^4^ Aspen Institute's Sports & Society Program Washington, D.C. USA

**Keywords:** disability, physical activity, youth sports

## Abstract

**Background:**

Although participating in youth sports benefits both youth with and without disabilities, those with disabilities are less likely to participate in organized sports than peers without disabilities and little remains known about the specific types of sports or the amount of time youth with disabilities engage. The present study is designed to address this gap, providing a descriptive understanding of the amount of time youth with disabilities play different sports and what type of sports these youth are participating in.

**Methods:**

A total of 1725 parents, guardians, and caregivers of youth athletes completed a national survey evaluating the state of youth sports in the United States, with 54.4% selecting that their child had a type of disability.

**Results:**

Using negative binomial regression, youth with disabilities play more days per week than youth without disabilities (*p* < 0.001), but average about one month less per year participating in their primary sport (*p* < 0.001). Using chi‐square test of independence followed by standardized residuals, youth with disabilities were more likely to play badminton (*Z* = −3.58), biathlon (*Z* = −2.43) and canoeing (*Z* = −2.25) and less likely to participate in soccer (*Z* = 2.53) and volleyball (*Z* = 2.46) compared with their peers without disabilities. When controlling for geographic region and disability type, additional differences in sport participation emerged.

**Conclusion:**

The distinct patterns in the types of sports that youth with disabilities participate in are likely influenced by multiple factors, including difficulties with physical access, lack of inclusion and increased time requirements. Because specific social and structural factors contribute to the types of sports youth with disabilities choose to participate in, it is worth continued exploration of participation trends, barriers and facilitators of sport involvement. Future studies should consider disability‐specific differences in sports participation, with emphasis on the amount and types of sports youth are playing.

## Summary

- Youth with disabilities may engage in less physical activity compared with youth without disabilities because they participate in sports fewer months per year.
- Youth with disabilities were found to participate in sports more days per week in the months they played their primary sport compared with youth without disabilities.
- Youth with disabilities likely participate in distinct sports compared with their peers without disabilities. Their specific type of disability and the region of the United States that they live in may further influence what sport they choose to participate in.
- Additional research is needed to evaluate the true participation trends of youth with disabilities, including from qualitative studies to understand the reasons for these trends in participation.

## 1 Introduction

Youth with disabilities are thought to engage in less physical activity and be less likely to play on a sports team than their peers without disabilities (Carbone et al. 2021; Ross et al. 2020; Jung et al. 2018; Kann et al. 2016; Martin Ginis et al. 2016; Woodmansee et al. 2016). There are many possibilities for why disparities in physical activity and youth sports exist and these reasons may be disability‐ and sport‐specific (Carbone et al. 2021; Martin Ginis et al. 2016; Haegele and Hodge 2017). However, there are likely some overarching trends, including difficulties with physical access or lack of adaptive equipment, lack of inclusion/feelings of inclusion and increased time requirements (Carbone et al. 2021; Zdroik et al. 2022; Okkenhaug et al. 2024; Shields and Synnot 2016; Wright et al. 2018). Despite the barriers, participating in youth sports can offer the same benefits for youth with and without disabilities (Carbone et al. 2021; Præst and Skovgaard 2022). The benefits of youth sports are far reaching, including both obvious physical activity benefits as well as supporting youth's psychosocial wellbeing (Carbone et al. 2021; Merkel 2013). Physical activity and youth sports can give positive long‐term health benefits, such as decreasing the possibility of developing some modifiable chronic diseases (e.g., type 2 diabetes mellitus, cardiovascular disease and certain cancers) (Merkel 2013). Youth with disabilities can also experience more specific benefits, especially when considering specific disability subtypes. For example, in one study, youth with intellectual/developmental disabilities participating in Special Olympics had higher self‐esteem, higher perceived abilities and greater peer acceptance compared with those who did not participate (Tint et al. 2017). Alternatively, among children and young people with physical impairments, sports are believed to lessen physical impairment and improve activity tolerance (West et al. 2025).

Given both the barriers and benefits of youth sports, researchers and physicians have called for more work to be done on the participation discrepancies present in youth sports (Carbone et al. 2021; Martin Ginis et al. 2016; Zdroik et al. 2022; Clutterbuck et al. 2025). This study seeks to provide much needed data on what types of sports youth with disabilities are participating in. Specifically, our first research question asks the following: how many months per year are youth with disabilities participating in their primary sport and, during those months, how many days per week are they playing? Our second research question asks the following: what is the primary type of sport that youth with disabilities are playing compared with their peers without disabilities? This question will be addressed broadly as well as considered in different regions of the United States and by disability types. We hypothesize that youth with disabilities will have higher participation rates in the four most common youth sports (basketball, soccer, baseball and tackle football) because the larger number of available teams increases the likelihood of finding options that are inclusive and accessible. Finally, our last question asks the following: how does the total number of sports played by youth with disabilities compare with youth without disabilities? Research currently suggests that youth with disabilities are less likely to participate in a sport; therefore, we hypothesize that, among youth with disabilities who play at least one type of sport, youth with disabilities play fewer types of sports compared with youth without disabilities (Ross et al. 2020). With these answers, we hope to address further what types of youth sports may be more or less inclusive and accessible and additional barriers to full access.

## 2 Method

This study is part of a larger study conducted to assess parent impressions of the state of youth sports in the United States. The study was approved by the Institutional Review Board of Utah State University and adhered to APA 7th edition ethical standards. Survey respondents were primary caregivers of youth who have participated in at least one sport in the past year. The survey was conducted via Qualtrics International, Inc. Respondents received $10 for their participation. Data were collected between 5 November and 19 December 2024. A total of 1725 respondents completed the survey; three were excluded from analysis because they indicated their child did not play any sports in the last year. Respondents were distributed across the United States and regions are based on the National Geographic Society's 2020 Breakdown of United States (see Table 1).

**TABLE 1:** United States geographic breakdown of home location of survey respondents.

Region	*N* (%)
Northeast	462 (26.8)
Southeast	442 (25.7)
Midwest	297 (17.2)
Southwest	160 (9.3)
West	361 (21.0)

The survey asked respondents about many aspects of their child's participation in youth sports. Respondents completed the survey for their oldest child, aged 6–18 years old, who participated in youth sports; average age of 11.57 (SD = 3.71) and 61.0% were identified as male (Table 2). Within the sample, 23.1% respondents selected ‘yes’ to their child having a documented disability. All respondents were then asked which disability(s) their child has; no disability was categorized as ‘other’ and none or N/A written in comments. The comments were reviewed and 24 responses were recategorized to a disability type based on the response in the comments and selection of questions. About 54.4% selected that their child had at least one of the disability types. The survey indicated that the most common diagnosis was ADHD (15.5%), followed by multiple disabilities (10.1%) and vision (6.5%; see Table 3). A total of 45.5% of respondents said their child had no disability.

**TABLE 2:** Demographic characteristics of youth with and without disabilities in this study.

	Youth with disabilities	Youth without disabilities
*N*	937	785
Age (SD)	11.2 (3.74)	12.1 (3.61)
Gender		
Male	581 (62.0%)	470 (59.9%)
Female	355 (37.9%)	314 (40.0%)
Nonbinary	1 (0.11%)	1 (0.13%)
Race/Ethnicity		
American Indian or Alaskan Native	13	8
Asian	32	27
Black or African American	200	164
Hispanic, Latino, or Spanish Origin	104	66
Native Hawaiian or Pacific Islander	5	0
White	529	467
Multiracial	48	42
Prefer not to say	2	2
Other	3	5
Annual income (SD)	$86,149 ($129,968)	$95,255 ($98,359)

**TABLE 3:** Breakdown of youth disability types reported in the survey.

Disability type	*N (%)*
Physical	96 (5.6)
Intellectual	46 (2.7)
Autism spectrum disorder	79 (4.6)
Attention deficit hyperactivity disorder	267 (15.5)
Vision	112 (6.5)
Hearing	27 (1.6)
Learning	102 (5.9)
Chronic disability	27 (1.6)
Other	8 (0.5)
Multiple disabilities	174 (10.1)
No disability	784 (45.5)

Respondents were then asked to select all of the sports that their child participated in over the past year, followed by what their child's primary sport is. Three respondents wrote in sports that were included in the list and were recategorized to the correct response. Six respondents selected only one sport in the past year and no primary sport, so were categorized with the one sport their child played in the past year. Next, respondents were asked the number of months during the year their child participated in their primary sport (options: 1–12 months). Respondents were then asked to select what formats their child was participating in youth sport: pick up or free play, individual practice or training, coach‐led practice or training, games or competition (options 1–7 days for each; can total more than 7 days in 1 week if participating in more than one category per day; Appendix A.1).

Data were stored on Box.com, an encrypted, cloud‐based platform. Poisson regression models with disability status as the main predictor were initially used to evaluate the first and third research questions: differences in months per year, days per week, total number of sports played. Because of evidence of overdispersion in the Poisson models, negative binomial regression was conducted as a sensitivity analysis to provide more robust estimates (*p* < 0.001 for all tests). Overdispersion likely existed due to the variety of sports that participant's played, with some sports having a limited number of participants (Appendix A.2). To examine the relationship between disability status and the type of sport played, a chi‐square test of independence was first used to evaluate youth participants in two groups: those who selected a disability type and those who did not. Sport type was treated as a categorical variable. In secondary analyses, standardized residuals were used to evaluate which sports contributed to the significant results. To control for potential confounding by region and disability type, we conducted multinomial logistic regression analyses with sport type as the outcome and disability status as the primary predictor, using the most common sport in the dataset, basketball, as the reference category. We additionally considered controlling for age, gender, race/ethnicity, and annual household income; however, models including all four demographic variables resulted in sparse cells and unstable parameter estimates and were therefore not retained for analysis. Given our research questions, we did not pursue separate models adjusting for each remaining demographic variable individually. All descriptive and inferential statistics were conducted in R studio (version 12.0+467), with significance set to *p* < 0.05 (R Core Team 2024).

## 3 Results

### 3.1 Sport Participation Frequency

Youth with disabilities played an average of 5.12 months per year (SD = 3.06) in their primary sport compared with 6.23 months per year (SD = 3.33) for youth without disabilities. This difference was statistically significant; negative binomial regression indicates that youth with disabilities participate 17.8% less than youth without disabilities (*IRR* = 0.82, 95% CI [0.78, 0.87], *p* < 0.001).

However, during the months when youth with disabilities played their primary sport, this group played more days per week than youth without disabilities overall (disabled: days = 9.25, SD = 6.20; nondisabled: days = 7.96, SD = 4.99). This is a statistically significant difference in the days played per group, with youth with disabilities playing on average of 16.0% more days per week than those without disabilities (*IRR* = 1.16, 95% CI [1.10, 1.23], *p* < 0.001). Youth with disabilities played more days of pick up or free play, individual practice or training, and games or competition. There was no difference in the number of days of coach‐led practice or training (see Appendix A.3).

### 3.2 Primary Types of Sports

There was a statistically significant difference in the sports played by youth with and without disabilities (*Χ* ^2^ = 88.65, *p* < 0.001). The effect size, as measured by Cramér's V, was 0.23, suggesting a small association according to the Cohen's thresholds (Cohen 1988). Standardized residuals showed that youth with disabilities were overrepresented in badminton (*Z* = −3.58), biathlon (*Z* = −2.43) and canoeing (*Z* = −2.25), whereas youth without disabilities were overrepresented in soccer (*Z* = 2.53) and volleyball, court or sitting (*Z* = 2.46; Table 4). When controlling for region, multinomial logistical regression showed no sports played more by youth with disabilities, while youth without disabilities played more cross country (OR = 1.74, 95% CI [1.19, 2.55], *p* < 0.001) and skateboarding (OR = 1.96, 95% CI [1.21, 3.16], *p* = 0.006; Table 4). When controlling for disability type, youth with disabilities were more likely to play artistic swimming (OR = 0.87, 95% CI [0.76, 0.99], *p* = 0.04) and biathlon (OR = 0.44, 95% CI [0.26, 0.75], *p* = 0.002; Table 4). No sports were played significantly more by youth with disabilities.

**TABLE 4:** Comparison between sport types.

Sport	Baseline	Controlling for region		Controlling for disability type	
	Disability (std. residual)^a^	OR^b^	*p*	OR	*p*
Archery	−0.69	0.97	0.87	0.93	0.26
Artistic swimming	−1.41	0.99	0.93	**0.87**	**0.04**
Badminton	−**3.58**	0.99	0.96	1.00	0.96
Baseball	−1.16	1.06	0.33	0.99	0.67
Basketball	0.25	Reference		Reference	
Bicycling	−1.08	0.91	0.63	1.08	0.40
Biathlon	−**2.43**	1.14	0.62	**0.44**	**0.002**
Bowling	−1.78	0.97	0.84	0.97	0.66
Bobsled, luge or skeleton	−0.43	0.88	0.77	0.81	0.23
Boxing	−0.57	1.21	0.23	1.07	0.43
Canoeing	−**2.25**	1.30	0.33	0.88	0.29
Cheerleading	−0.05	1.10	0.32	1.02	0.73
Cross country	1.95	**1.74**	**0.005**	1.17	0.14
Curling	−0.92	0.70	0.67	1.07	0.85
Dance	0.02	1.04	0.63	1.02	0.57
Diving	−0.83	1.52	0.22	1.20	0.37
Equestrian	−0.25	0.91	0.78	0.88	0.33
Field Hockey	0.65	0.72	0.38	1.00	0.97
Figure Skating	−1.83	1.08	0.81	0.94	0.66
Football (flag)	−1.45	1.06	0.58	0.95	0.23
Football (tackle)	1.88	1.08	0.24	1.04	0.20
Golf	−0.75	1.25	0.22	1.13	0.22
Gymnastics	1.92	1.20	0.06	1.07	0.15
Ice hockey	−0.37	0.95	0.76	0.96	0.53
Judo	−0.43	0.88	0.77	1.31	0.34
Karate/mixed martial arts	0.71	1.14	0.26	1.10	0.12
Lacrosse	−0.52	1.25	0.23	0.94	0.46
Parkour	1.09	412.36	0.83	1.91	0.60
Pentathlon	−0.92	412.85	0.83	0.51	0.30
Pickleball	−0.43	3.60	0.09	0.99	0.96
Rock climbing	−1.59	1.21	0.62	0.69	0.09
Roller skating	−0.92	1.21	0.77	0.81	0.50
Rowing	−0.43	1.41	0.38	0.78	0.18
Rugby	0.73	0.00	0.93	1.19	0.45
Shooting sports	−1.59	0.88	0.77	1.14	0.54
Skateboarding	−1.63	**1.96**	**0.006**	0.84	0.07
Skiing/snowboarding	1.86	1378.59	0.74	1.14	0.39
Soccer	**2.53**	1.06	0.32	1.03	0.23
Softball	1.22	0.85	0.22	1.02	0.67
Speedskating	−0.92	0.70	0.67	0.52	0.29
Surfing	−0.92	412.27	0.83	7.10	0.33
Swimming	0.02	1.23	0.08	0.99	0.80
Table tennis	0.73	1.04	0.92	1.03	0.89
Tennis	0.68	0.91	0.47	1.04	0.53
Track & field	0.15	1.02	0.87	1.03	0.66
Ultimate Frisbee	−0.92	1.21	0.78	0.71	0.35
Volleyball (beach)	−1.44	0.76	0.25	1.05	0.62
Volleyball (court or sitting)	**2.46**	1.18	0.08	1.07	0.15
Water polo	−1.59	1.04	0.92	0.81	0.23
Weightlifting	−0.009	1.37	0.13	1.04	0.73
Wrestling	−0.46	1.09	0.53	0.99	0.81
Other	−1.08	1.21	0.30	1.02	0.80

*Note:* Bold = significant result.

^a^ Youth without disabilities were set as the reference. Positive values were overrepresented by youth without disabilities and negative values were overrepresented by youth with disabilities.

^b^ Certain sports had no participants either with or without a disability, leading to inflated odds ratio estimates. These findings were therefore interpreted with caution and considered in the context of the sport‐specific sample sizes.

### 3.3 Total Number of Sports Played

Youth with disabilities played an average of 2.82 different sports (SD = 2.93) and youth without disabilities played an average of 2.46 different sports (SD = 2.04). There was a statistically significant difference in the total number of sports played (IRR = 1.14, 95% CI [1.06, 1.23], *p* < 0.001).

## 4 Discussion

Across the three research objectives, our findings suggest that there are differences in amount and types of sports participation between youth with and without disabilities nationally, regionally, and when examining different types of disabilities across the United States.

The first research objective considered how many months per year and days per week youth with disabilities were participating in their primary sport. We found that youth with disabilities play their primary sports for fewer months out of the year (disabled: months = 5.12; nondisabled: months = 6.23). However, during those months, youth with disabilities are playing more days per week (disabled: days = 9.25; nondisabled: days = 7.96). Prior research has reported mixed findings regarding physical activity participation among youth with disabilities compared with their peers without disabilities (Ross et al. 2020; Jung et al. 2018). Ross et al. (2020) found that children with disabilities engaged in less physical activity than their nondisabled peers, whereas adolescents with and without disabilities participated at similarly low levels. Similarly, Jung et al. (2018) reported comparable participation in low‐intensity physical activity between youth with and without disabilities, but lower participation among youth with disabilities in moderate‐ to vigorous‐intensity physical activity. Among youth with disabilities who do sports, this group is more likely to meet their physical activity goals (Ross et al. 2020). While there are multiple avenues for youth to participate in physical activity, our study suggests that lower physical activity may in part be due to youth with disabilities playing sports fewer months out of the year, but equal or greater participation when their sport is in session.

When youth with disabilities are participating in their primary sport, our study found that youth with disabilities may participate more in the settings of free play, individual practice and games or competition. Interestingly, there was no significant difference in the number of days of coach‐led practices (Appendix A.3). These findings suggest that greater sport participation among youth with disabilities may occur outside of structured practices. Previous research has shown that sport participation may provide greater benefits for youth with disabilities, including higher self‐esteem, improved socio‐emotional adjustment and greater perceived competence (Carbone et al. 2021; Tint et al. 2017; Bohnert et al. 2019). These benefits may encourage youth with disabilities to participate more outside of organized practices, although the relationship is likely complex in nature.

Our second research question asked what types of sports that youth with disabilities are participating in, generally and when controlling for disability type and region. We hypothesized that youth with disabilities will have higher participation rates in the four most common youth sports (basketball, soccer, baseball and tackle football). Our findings contradicted this hypothesis, suggesting that youth with disabilities are more likely to play badminton, biathlon and canoeing and less likely to participate in soccer and volleyball, although the effect size was small. However, these findings should be interpreted with caution given small sample sizes in certain sports. There are many possible explanations for this finding, such as sport‐specific cultures, physical access challenges, cost or negative attitudes (Carbone et al. 2021; Martin Ginis et al. 2016; Haegele and Hodge 2017). Woodmansee et al. (2016) found that youth with disabilities participated less frequently in team sports, more frequently in swimming, and reported lower enjoyment of individual physical activities. Our findings partially align with these results, as youth with disabilities were less likely to participate in certain team sports, including soccer and volleyball. However, they were more likely to participate in sports such as badminton, biathlon and canoeing, which are typically individual or small‐group activities. Together, these findings suggest that sport participation patterns among youth with disabilities are complex but may reflect an overall trend toward lower participation in team sports.

When controlling for disability type, youth with disabilities were more likely to participate in artistic swimming and biathlon. Whereas when controlling for region, youth without disabilities were more likely to play cross country and skateboarding. Type of disability and/or United State region may, therefore, explain the difference in participation across the different sports. This interpretation is consistent with previous research by Ross et al. (2020), which found that youth with cognitive, mobility and functional impairments were among the least likely to achieve recommended levels of physical activity, with youth with cognitive and mobility impairments also less likely to participate in organized sports. Together, these findings suggest that sport participation among youth with disabilities is influenced not only by disability type but also by regional differences in access to and availability of sport opportunities.

Our third research objective evaluated the number of sports youth with and without disabilities play, finding that youth with disabilities play more different sports than youth without disabilities (disabled: sports = 2.82; nondisabled: sports = 2.46). Despite statistical significance, the value and standard deviations showed considerable overlap, suggesting that additional research is needed to confirm whether a true difference exists in the number of sports youth with and without disabilities are participating in. Previous research suggests differences in sport selection, while your findings suggest these differences do not necessarily translate to fewer sports overall (Woodmansee et al. 2016).

This study provides much needed data on the amount and types of sports youth with disabilities participate in. The strengths of this study include the large sample size, representing diverse youth across all 50 United States. Given the immense benefits of youth sport participation, it is critical to continue to promote youth sports and better understand the types of sports youth with disabilities are participating in.

## 5 Limitations

While our study is novel in many respects, there are limitations that should be acknowledged. First, using the biopsychosocial model of disability, disability can be a contextualized and individualized experience (World Health Organization 2001). A total of 54.4% of our respondents selected a type of disability, but only 23.1% selected that their child had a documented disability. It is unclear why 31.3% of respondents selected a type of disability for their child but did not have a documented disability. For the purpose of this study, those who selected a disability type(s) were included as having a disability. Future research should seek to operationalize their definitions of disability within the survey to better capture true numbers of youth with and without disabilities.

Furthermore, 10.1% of respondents selected multiple types of disability. When controlling for type of disability, these respondents were included in a category of ‘multiple disabilities’. However, this fails to understand the unique barriers that youth with multiple disabilities face when participating in youth sports. Future research must consider the ways in which researchers discuss and ask about disability in surveys.

Within certain types of sports, there was also limited data with very few youth with or without disabilities participating in certain sports (see Appendix A.2). While disability type was included as a covariate in our analyses, small sample sizes within individual disability categories limited our ability to separately model sport participation patterns by disability type. This is an important area for future research, as different disability types may influence sport participation through distinct physiological, cognitive, or environmental factors. Additionally, although age, gender, race/ethnicity and annual household income have been seen to influence sport participation, including all four variables simultaneously as controls resulted in sparse cell counts and unstable parameter estimates. These variables were not included as controls in the current analysis to maintain focus on disability‐related and geographic differences in participation (Ross et al. 2020). Future studies with larger samples should consider examining how demographic factors interact with disability status to influence sport participation.

Finally, our survey results are based upon parent or caregiver reported data. While this study most directly considered only quantitative, nominal markers, we hypothesized on reasons for why disparities in youth sports may exist. This study makes it clear that there are differences in sports participation that are unclear or unknown. The only way to learn these answers is to engage with youth with disabilities about barriers and successes in these spaces. Alongside other researchers, we call for youth with disabilities to be included in the discussion and research of youth sports (Maher and Haegele 2022).

## 6 Conclusion

Youth with disabilities are thought to get less physical activity and be less likely to play on a sports team than their youth without disabilities (Carbone et al. 2021; Ross et al. 2020; Jung et al. 2018; Kann et al. 2016; Martin Ginis et al. 2016; Woodmansee et al. 2016). The reason for this disparity remains complex, yet it remains important to understand the contexts in which youth with disabilities are participating. Our study found quantitative differences in the amount of months per year, days per week, number of different sports and avenue of sports participation, with youth with disabilities playing fewer months per year in their primary sports, but more days per week. Further, the study found that certain sports (badminton, biathlon and canoeing) may be more commonly played by youth with disabilities with possible explanations for this finding being due to a youth's disability type or United States region. Given the widespread benefits of youth sport, our findings showcase possible participation differences among youth with and without disabilities, warranting further evaluation into the true participation of youth with disabilities in sports (Carbone et al. 2021; Præst and Skovgaard 2022).

## Author Contributions

**Natalie H. Stagnone:** formal analysis, writing – original draft, writing – review and editing. **Andrew Parks:** conceptualization, writing – review and editing. **Travis E. Dorsch:** conceptualization, methodology, data curation, supervision, funding acquisition, writing – review and editing. **Jordan A. Blazo:** conceptualization, methodology, data curation, supervision, funding acquisition, writing – review and editing. **Tom Farrey:** conceptualization, methodology, data curation, supervision. **Jon Solomon:** conceptualization, methodology, data curation, supervision. **Jennifer Brown Lerner:** conceptualization, methodology, data curation, supervision.

## Funding

This study was funded by The Aspen Institute Sports & Society Program.

## Disclosure

The authors of this manuscript confirm that no copyrighted material belonging to third parties has been reproduced, adapted, or incorporated in any form within this work. All text, figures, tables, data, and other content presented in this manuscript are original contributions of the authors. Therefore, no permission letters or licensing agreements are applicable to this submission.

## Ethics Statement

The study was approved by the Institutional Review Board of Utah State University and adhered to APA 7th edition ethical standards.

## Consent

Informed consent for publication was obtain for all participants in accordance with Institutional Guidelines at Utah State University. A copy of the consent form is available for review by the editor of this journal.

## Conflicts of Interest

The authors declare no conflicts of interest.

## Data Availability

Data from this project are proprietary, co‐owned by the authorship team and then funding partner, and will not be shared publicly.

## Appendix A

### A.1 Survey Items Used in This Study

Survey Item: In what state does your family currently reside?

Response Options: All United States.

Survey Item: What is your family's ANNUAL INCOME across all earners, before taxes?

Response Options: Numeric Value

Survey Item: What is your child's AGE?

Response Options: Numeric Value

Survey Item: What is your oldest child's SEX?

Response Options: Male, Female, Non‐binary

Survey Item: What is your child's RACE or ETHNICITY?

Response Options: American Indian or Alaskan Native, Asian, Black or African American, Hispanic, Latino, or Spanish Origin, Native Hawaiian or Pacific Islander, White, Multiracial, Prefer not to say, Other

Survey Item: Does your child have any documented DISABILITIES?

Response Options: Yes, No

Survey Item: Which of the following disabilities does your child have?

Response Options: Physical, Intellectual, Autism Spectrum Disorder (ASD), Attention Deficit Hyperactivity Disorder (ADHD), Vision, Hearing, Learning, Chronic Disease, Other

Survey Item: Please select the sport(s) in which your child participated and/or competed

DURING THE LAST YEAR.

Response Options: List of sports

Survey Item: Of the sports you just identified, which would you consider your child's PRIMARY SPORT?

Response Options: List of sports

Survey Item: How many MONTHS DURING THE YEAR does your child participate in scheduled practices or competitions in their primary sport?

Response Options: 1‐12 month selection option

Survey Item: When your child's primary sport is in‐season, how many DAYS PER WEEK do they engage in the following types of participation in that sport?

Response Options: Pickup or free play, individual practice or training, coach‐led practice or training, games or competition

### A.2 Sports Played by Youth With and Without Disabilities

**Table:**

Sport	Number of youth	
	Disability	No Disability
Archery	13	8
Artistic swimming	14	6
Badminton	26	4
Baseball	103	73
Basketball	209	179
Bicycling	9	4
Biathlon	7	0
Bowling	18	7
Bobsled, luge or skeleton	2	1
Boxing	11	7
Canoeing	6	0
Cheerleading	29	24
Cross country	4	10
Curling	1	0
Dance	38	32
Diving	3	1
Equestrian	3	2
Fencing	0	0
Field hockey	2	3
Figure skating	4	0
Football (flag)	34	19
Football (tackle)	62	71
Golf	9	5
Gymnastics	22	31
Ice hockey	10	7
Judo	2	1
Karate/mixed martial arts	18	19
Lacrosse	8	5
Parkour	0	1
Pentathlon	1	0
Pickleball	2	1
Rock climbing	3	0
Roller skating	1	0
Rowing	2	1
Rugby	1	2
Shooting sports	3	0
Skateboarding	8	2
Skiing/snowboarding	1	5
Soccer	113	128
Softball	15	19
Speedskating	1	0
Squash	0	0
Surfing	1	0
Swimming	19	16
Table tennis	1	2
Tennis	15	16
Track & field	17	15
Ultimate Frisbee	1	0
Volleyball (beach)	9	3
Volleyball (court or sitting)	21	34
Water polo	3	0
Weightlifting	6	5
Wrestling	17	12
Other	9	4

### A.3 Bar Chart of Days Played Per Week by Types of Participation in Youth's Primary Sport

Total number of days is the sum of other categories. It may exceed seven days per week because youth are participating in more than one category in a single day.
